# Progress in Psychiatry

**Published:** 1904-05-07

**Authors:** 


### PROGRESS IN PSYCHIATRY.

*Concluded from page 60.*

**Alcoholism and Suicide.**—In a recently published article,[11] Dr. W. C. Sullivan, of His Majesty's Prison, Pentonville, attempts to determine the rôle which alcoholism plays in the increase of suicide in England, and to establish the distinctive characters which constitute the type of alcoholic suicide. Dr. Magnus Huss, in his classical work on Chronic Alcoholism (1852), stated that the suicidal impulse is a more frequent accompaniment of the melancholia of drunkards than of melancholia from other causes, and, further, that among the uneducated classes suicide frequently follows on the disordered emotional tone which sooner or later results from the abuse of alcoholic liquors." The majority of observers who have studied the question of suicide—Morselli, Wynn Westcott, Ferri—argue that the two phenomena of alcoholism and suicide show sufficiently close correspondence in regard to geographical distribution and time to justify the inference of their relationship as cause and effect. After quoting the Registrar-General's returns of the deaths attributed to intemperance (1867–1897) and the figures in the memorandum (by Mr. Whittaker), published with the report of the Licensing Commission, which gives the consumption of beer and spirits in the United Kingdom for the years 1892–1898, Dr. Sullivan concludes from a careful study of the facts and figures that they represent a real and decided increase of alcoholism in this country of late years. The statistical returns of actual and of attempted suicide are next discussed, and curves are plotted from which it appears "that suicidal tendencies have grown in a degree entirely out of proportion to the increase in population," and that this growth has been much more considerable in the category of suicidal attempts than in that of actual suicides. It also appears that abortive suicidal attempts differ widely from actual suicides in that their predominant cause tends to operate at a relatively early age, the period of 21 to 30 years of age in females and of 30 to 40 years in males being the maxima. Now the tendency to occur at a relatively early age, which is the chief distinctive feature of abortive suicidal attempts, is also the character of an actual group of suicides—viz. those dependent upon alcoholism. In the groups of occupations dealt with in the Registrar-General's returns there are several which present a very high rate of alcoholism and a corresponding frequency of suicide. These are more particularly the groups of occupations related to the liquor traffic, or those where social conditions lead directly to alcoholic excess—*e.g.* publicans, butchers, the coach and cab service, commercial travellers, and musicians. On the other hand, the agricultural occupations furnish a low proportion of cases both of alcoholism and of suicide. The explanation of the frequency of the suicidal tendency in the alcoholic is that the visceral and organic depression and consequent tendency to melancholia resulting from alcoholism are favourable to the evolution of suicidal feelings, and, moreover, that suicidal impulses arising from this or any other cause during intoxication are not combated or overcome by the depressed and melancholic victims of chronic alcoholism, whereas in healthy subjects such impulses when they arise are speedily overcome.

**Alcoholism and Homicide.**—In a further contribution[12] to the same journal, Dr. Sullivan deals with the relations of alcoholism and homicide. The paper is based on a series of 80 cases, comprising 36 cases of homicide and 44 of grave homicidal attempts, "in which the criminal act could in some considerable measure be assigned to the influence of alcoholism." Several cases are given, of which the following one may be cited as a typical case. The patient was a man aged 28 years, by occupation a sailor. His father was mentally unstable and "subject to fits." The patient's paternal uncle died insane, and the patient himself is said to have had sunstroke. When sober he was a man of normal intelligence and feelings, but when intoxicated he was violent. On the evening of the 11th day of a severe drinking bout, he was seen to go home with his wife, and appeared to be on "boisterously affectionate" terms with her. During the night he cut her throat with a razor as she lay in bed, and then made an almost successful attempt to commit suicide with the same weapon. He professed to have no memory of the act, and could not at any subsequent time suggest a motive for it. While under treatment for his self-inflicted wound he suffered from the severe digestive and nervous symptoms of alcoholism. This was, says Dr. Sullivan, a good example of the "automatic type of alcoholic homicide. As in the corresponding form of alcoholic suicide [referred to in the previous paragraph] there is an entire absence of apparent motive, the act is committed in a state of intoxication, no trace is left in the agent's memory of the crime, and finally, to show the identity in nature and origin of the two impulses, the murder is followed by a suicidal attempt." In explanation of this it is pointed out that the cerebral condition in intoxication is one of excitability of the kinæsthetic or so-called "motor" areas and undue suggestibility. Under these conditions a slight "suggestion," arising from whatever cause, within the mind, tends to be translated into action with unrestrained facility. A clear instance of such an agency, trivial in itself but potent in the peculiar case of alcoholic persons, is afforded in a case recorded by Professor Despine, where one of four drunkards who were carousing together suggested the hanging of the most intoxicated one of the party—a suggestion which was promptly carried out, and only failed of being fatal through the accident of outside intervention. In other alcoholic subjects the murder of a wife may be the result of delusions of marital infidelity or of poisoning, for such delusions are very prone to develop in chronic alcoholism. Other chronic alcoholics are liable to attacks of mental disturbance of an epileptic character with a condition of mental automatism succeeding the attack, in which state murder may be unconsciously committed and no memory retained of the act. In few cases is there any degree of lucidity and purposiveness in the homicidal acts of the chronic alcoholic. Chronicity and long indulgence in the drinking habit are the natural antecedents of true alcoholic homicide, except in cases where there exists initially some degree of mental instability, hereditary or acquired. There may then be a very precocious development of impulsive automatism in which the rôle of intoxication is largely overshadowed by the primary mental abnormality or disorder. Cases of this kind form the transition to insane homicide unconnected with alcoholism.

[11] Jour. of Ment. Sci., April, 1900. [12] Ibid., Oct., 1900.

